# Thoracic kyphosis in light of lumbosacral alignment in thoracic adolescent idiopathic scoliosis: recognition of thoracic hypokyphosis and therapeutic implications

**DOI:** 10.1186/s12891-022-05379-6

**Published:** 2022-05-03

**Authors:** Takuya Iimura, Haruki Ueda, Satoshi Inami, Hiroshi Moridaira, Daisaku Takeuchi, Hiromichi Aoki, Hiroshi Taneichi

**Affiliations:** Department of Orthopaedic Surgery, Dokkyo Medical University, 880 Kitakobayashi, Mibu, Tochigi, Shimotuga 321-0293 Japan

**Keywords:** Thoracic AIS, Sagittal alignment, Hypokyphosis, Sacral slope, Max-LL, Inflection point

## Abstract

**Background:**

The uniqueness of spinal sagittal alignment in thoracic adolescent idiopathic scoliosis (AIS), for example, the drastically smaller thoracic kyphosis seen in some patients, has been recognized but not yet fully understood. The purpose of this study was to clarify the characteristics of sagittal alignment of thoracic AIS and to determine the contributing factors.

**Methods:**

Whole spine radiographs of 83 thoracic AIS patients (73 females) were analyzed. The measured radiographic parameters were the Cobb angle of thoracic scoliosis, thoracic kyphosis (TK), lumbar lordosis (LL), C7 sagittal vertical axis (C7 SVA), pelvic incidence (PI), pelvic tilt (PT), and sacral slope (SS). Additionally, max-LL, which was defined as the maximum lordosis angle from the S1 endplate, the inflection point between thoracic kyphosis and lumbar lordosis, and the SVA of the inflection point (IP SVA) were measured. The factors significantly related to a decrease in TK were assessed by stepwise logistic regression analysis. In addition, cluster analysis was performed to classify the global sagittal alignment.

**Results:**

The significant factors for a decrease in TK were an increase in SS (*p* = 0.0003, [OR]: 1.16) and a decrease in max-LL (*p* = 0.0005, [OR]: 0.89). According to the cluster analysis, the global sagittal alignment was categorized into the following three types: Type 1 (low SS, low max-LL, *n* = 28); Type 2 (high SS, low max-LL, *n* = 22); and Type 3 (high SS, high max-LL, *n* = 33).

**Conclusions:**

In thoracic AIS, a decreased TK corresponded to an increased SS or a decreased max-LL. The sagittal alignment of thoracic AIS patients could be classified into three types based on SS and max-LL. One of these three types includes the unique sagittal profile of very small TK.

## Background

Along with the developing interest in global spinal balance, many studies have delineated the normal spinopelvic sagittal alignment of healthy adults, adolescents, and children in standing posture. When normal individuals stand, their global sagittal balance is maintained under a reciprocal and harmonic relationship between the spinal and pelvic parameters, although the parameters in children and adolescents are different from those in adults; that is, younger populations have smaller pelvic incidence, pelvic tilt, and thoracic kyphosis parameters and larger lumbar lordosis [1, 2]. However, this topic has not yet been studied in scoliotic adolescents. Adolescent idiopathic scoliosis patients with a major thoracic curve (thoracic AIS) often present with unique sagittal alignment, including hypokyphosis in the thoracic spine, causing cosmetic, self-image, and pulmonary functional problems [3, 4]. Hypokyphosis after scoliosis surgery for thoracic AIS may not affect pain or mental status on QOL questionnaires in the short term postoperatively [5], but an increasing number of studies have reported that postoperative hypokyphosis in this population could result in problems in radiographic parameters, including lumbar disc degeneration, cervical decompensation, deterioration of sagittal vertical axes, and proximal junctional kyphosis, which could lead to clinical issues in the future [6–9]. Therefore, adequate attention to the sagittal profile is certainly critical to achieving better treatment outcomes in thoracic AIS. The unique sagittal profile of this condition should be scrutinized, but reports on this topic are scarce [10, 11]. The purpose of this study was to clarify the characteristics of the global and thoracic sagittal alignment of thoracic AIS.

## Methods

This is a retrospective radiographic survey in a single institute. Whole-spine X-ray films acquired of thoracic AIS (Types 1 and 2 in the Lenke classification) patients while standing were analyzed. Only films that clearly visualized the whole spine and bilateral femoral heads were included. Films were excluded if they could not be clearly measured or if they showed anomalies in the pelvis or hips and/or a leg length discrepancy. A total of eighty-three patients (10 males and 73 females) with an average age of 15.9 years (10–26 years) were enrolled and analyzed. This study was approved by our institutional review board (approval number 29018).

### Radiographic measurements

**Fig. 1:**
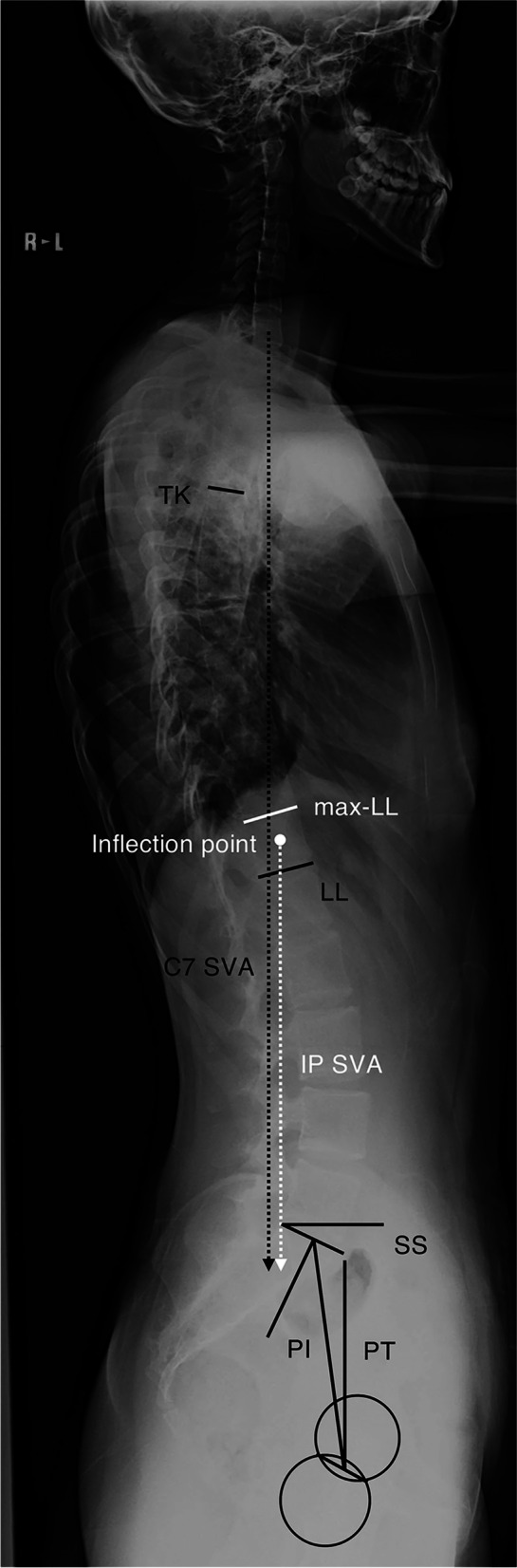
Radiographic parameters were measured on lateral images of the whole spine while the patients were standing. Max-LL: maximum lordotic Cobb angle from S1, IP: inflection point between thoracic kyphosis and lumbar lordosis, IP SVA: sagittal vertical axis of IP

For all patients, standing posteroanterior (PA) and lateral radiographs of the whole spine and pelvis were pooled in our PACS system (CentricityTM Enterprise Web, version 3.0. GE Healthcare Japan, Tokyo), and all radiographic measurements were digitally performed on Surgemap 2.0.6 software (Nemaris, Inc. NY. USA). The measured radiographic parameters were as follows (Fig. 1): Cobb angle of thoracic scoliosis (Thoracic Cobb), thoracic kyphosis (TK; angle between the superior end plate of T5 and inferior end plate of T12), lumbar lordosis (LL; angle between the inferior end plate of T12 and superior end plate of S1), C7 sagittal vertical axis (SVA), pelvic incidence (PI), pelvic tilt (PT), and sacral slope (SS). Additionally, max-LL was defined as the maximum lordotic Cobb angle from S1 and was measured from the upper end plate of S1 to the upper endplate of the vertebra at the inflection point between thoracic kyphosis and lumbar lordosis. This inflection point was also recorded and denominated 0 when this point was at L1 and assigned a positive number when this point descended caudally (i.e., + 1 at L2, + 2 at L3, -1 at T12). The SVA of this inflection point (IP SVA) was measured. Both SVAs (C7 SVA, IP SVA) were measured as the horizontal distance from the posterosuperior corner of the S1 vertebral body.

### Statistical analysis

To determine the significant factors related to a decrease in TK, stepwise logistic regression analysis was conducted with TK as an objective variable and the other measured parameters as explanatory variables. With the significant factors identified by the stepwise regression analysis, cluster analysis was performed to classify the global sagittal alignment. Analysis of variance and multiple comparisons were used to compare the parameters between the types classified by cluster analysis. A *p* value < 0.05 was considered statistically significant for all tests. All analyses were performed by JMP 10.0.2 software (SAS Institute Inc.)

## Results

**Fig. 2:**
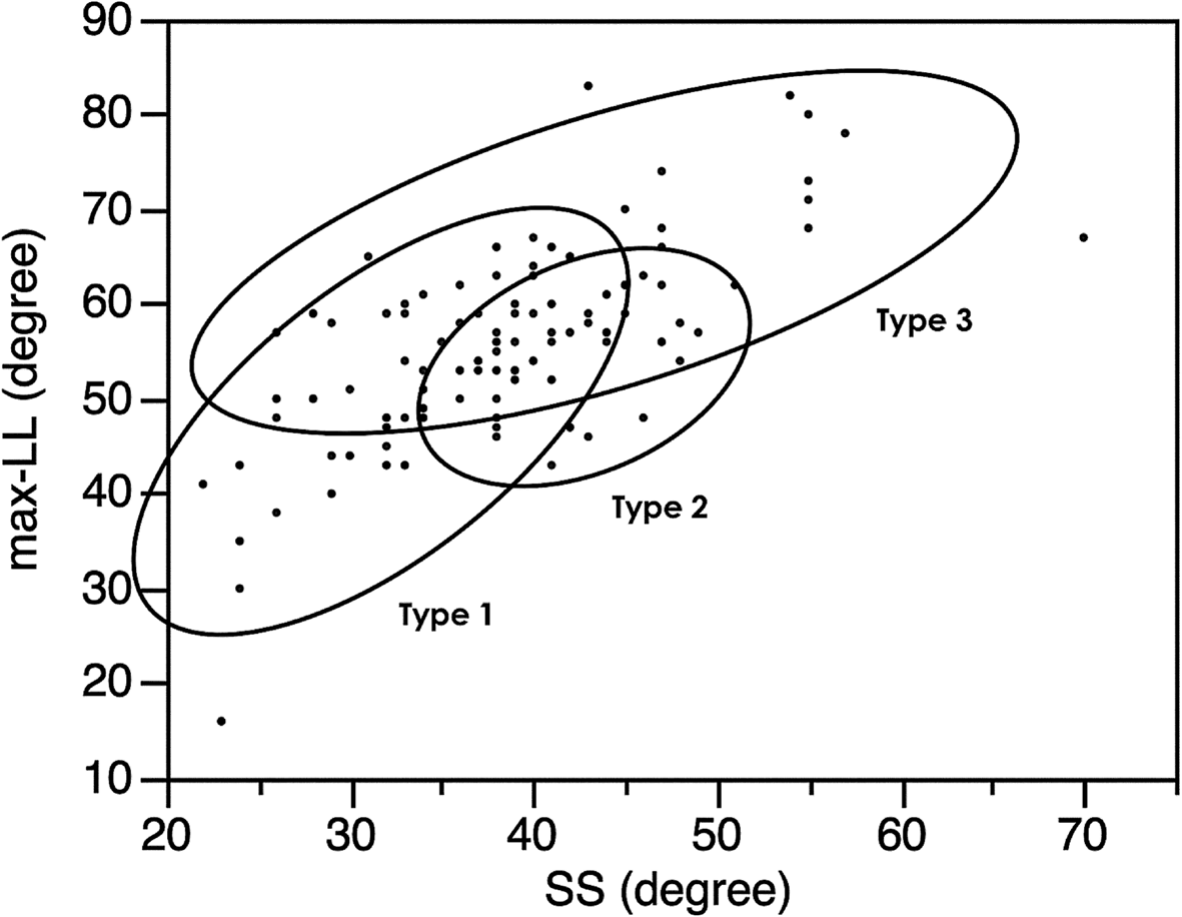
Cluster analysis of sagittal alignment in thoracic AIS. Cases were classified into three types based on their SS and Max-LL

**Fig. 3:**
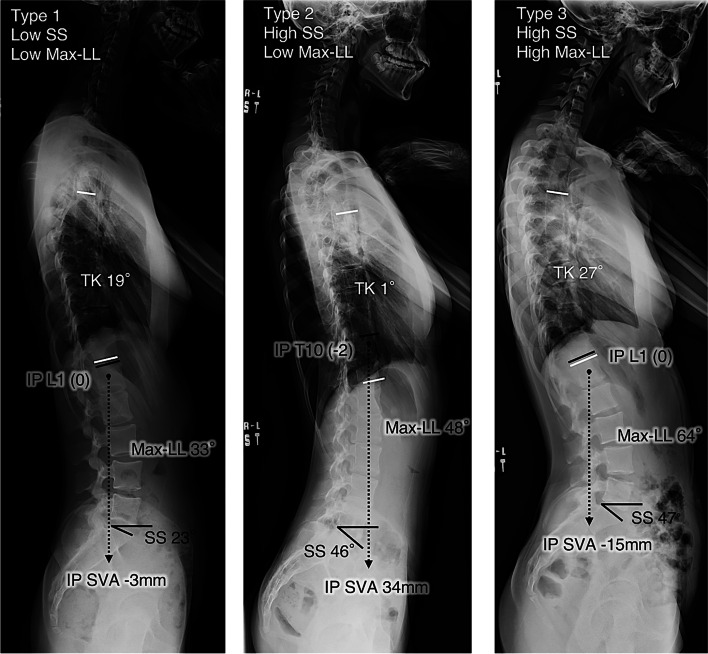
Lateral X-ray films of representative cases. Type 1 is generally manifests with a flat sagittal profile with a smaller SS and Max-LL. Type 2 demonstrates a larger SS, small Max-LL and a significantly small TK. Type 3 shows a large SS and Max-LL

**Fig. 4:**
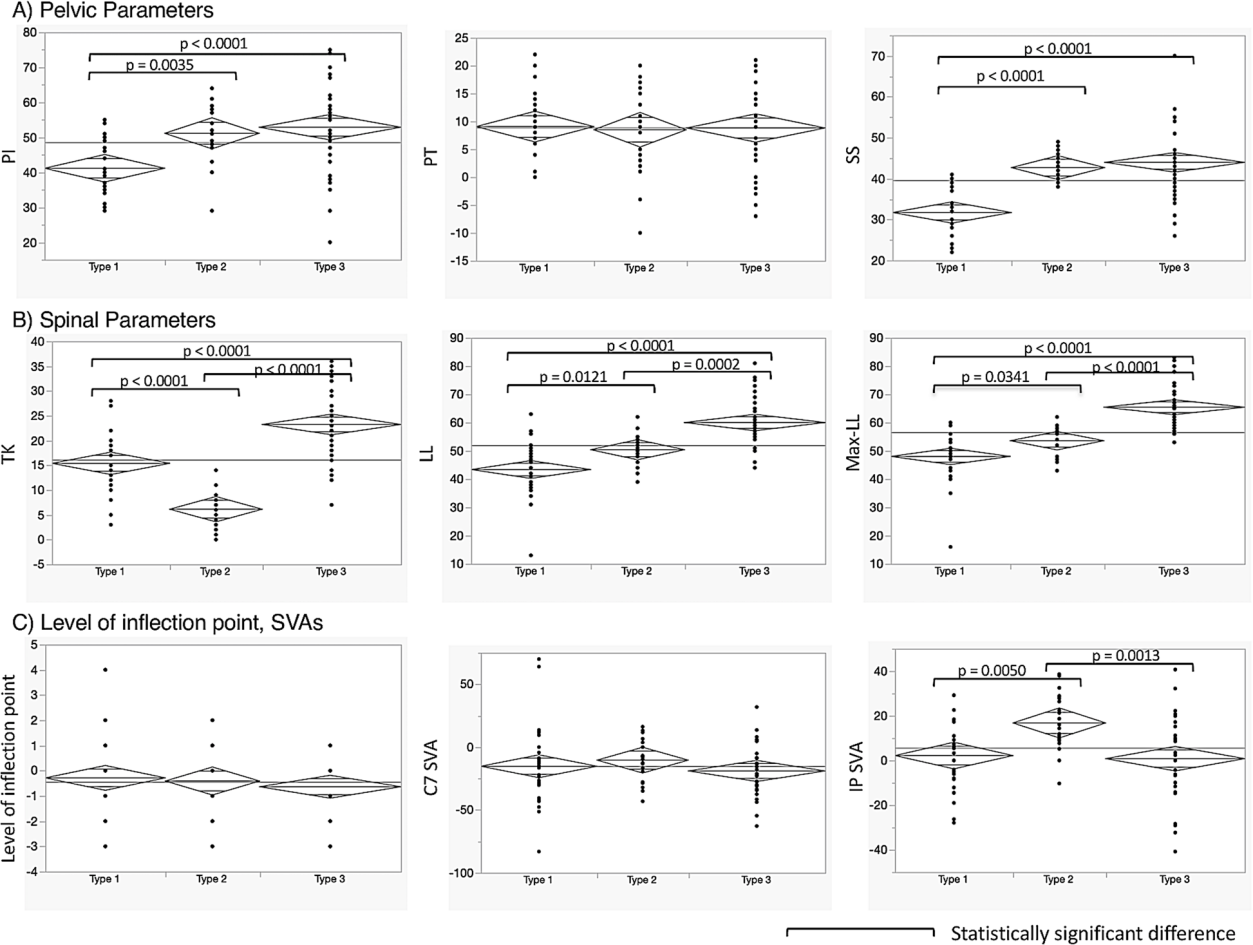
The comparison of radiographic parameters between three types. (One-way ANOVA, JMP 10.0.2 software) **A** Pelvic parameters. Type 1 has outstandingly small PI and SS values compared to the other types. **B** Spinal parameters. These are all significantly different among the three types. **C** Levels of inflexion points and SVAs. Type 2 has a significantly anterior IP SVA compared to the other types. Despite their similar PI and SS measurements, type 2 and type 3 have significantly different TK, LL, max-LL, and IP SVA measurements

All the measured parameters are summarized in Table 1. Based on stepwise regression analysis, max-LL, SS, and IP SVA were selected as the explanatory variables of small TK. Then, multivariate logistic regression analysis was conducted with these three selected variables, and max-LL and SS were identified as significant risk factors for small TK (Table 2). Using SS and max-LL, cluster analysis was performed and showed that thoracic AIS can be classified into the following three types in terms of global sagittal alignment (Fig. 2): Type 1: low SS with low max-LL; Type 2: high SS with low max-LL; and Type 3: high SS with high max-LL. Representative cases are presented in Fig. 3. Comparisons of each parameter between the three types is shown in Fig. 4. The average PI and SS in Type 1 were significantly smaller than those in the other types. A significantly small TK and large IP SVA are distinctive of Type 2. Despite similar PI and SS values, Types 2 and 3 have significantly different LL and max-LL values. The average inflection point in all three types was between -0.3 and -0.6, meaning between T12 and L1 in all types.

## Discussion

Ever since the importance and influence of spinopelvic alignment on health-related quality of life was recognized, avid research has deepened our understanding of the normal values and pathophysiology related to abnormal alignment. This topic has not been scrutinized in the adolescent scoliosis population, who have a very unique sagittal profile due to the abnormal curve and rotation of the spine.

Thoracic AIS has been reported to have small TK. This trend can be universally found in black, white, and Asian populations, but especially in the Asian population [10, 12–15]. Among AIS patients, those with a primary curve in the thoracic spine had smaller TK than those with a primary curve in the lumbar spine [13, 14]. In our study, in which all patients were Japanese, the average TK was as low as 16.1°, which, interestingly, is close to that reported in Chinese girls with T-AIS by Yong (Table 3) [10].

Clement found that half of his included cohort had very small TK, with an average of 8.2° [12]. Similar to his report, a cluster of patients in our cohort had very small TK and was classified as Type 2. These patients are reported to be at risk for several clinical issues, including pulmonary impairment, lumbar disc degeneration, and possibly neck pain, many years after fusion [3, 4, 6, 16–18]. In particular, the negative impact of thoracic hypokyphosis on pulmonary function has been considered clinically important and requires the appropriate choice of treatment. In this regard, Winter et al. recommended in his early case report on thoracic lordoscoliosis that these patients should undergo early fusion surgery rather than continue brace treatment if the lordosis increases under brace treatment [19]. The flattening effect of brace treatment for AIS patients on the sagittal profile has been reported, and this effect from orthotics understandably exerts a negative impact on primarily hypokyphotic patients with decreased pulmonary function [20, 21]. Hence, for this group of patients, earlier surgical correction should be considered with the objective to restore thoracic kyphosis. Several techniques have been introduced for the correction of thoracic kyphosis, including meticulous facetectomy for spinal column release, surgery with a higher implant density and a pedicle screw system, and the vertebral coplanar alignment method [22, 23].

The correlation between lumbar and sacropelvic alignment has been advocated for in various populations, including healthy children, healthy adults, and scoliotic adult scoliotic patients [1, 2, 24]. Thoracic AIS can be expected to have some correlation to lumbosacropelvic parameters, similar to that seen in the normal population. The correlation between thoracic and sacropelvic parameters remains unclear. However, this topic is reported less clearly, and these parameters seem to have weaker correlations with each other. The spinopelvic parameters of healthy children in a similar age group to those in this study were reported as follows: TK 20.8 ~ 46°, LL 48 ~ 57°, PI 44.6 ~ 46.9°, PT 7.7 ~ 11.3°, and SS 33.3 ~ 39.1° [2, 10, 25–27]. Compared to these values, the parameters in this study were all similar except for TK, which was smaller. This can be interpreted as thoracic scoliosis influencing only TK in the sagittal spinopelvic parameters. This is consistent with a report of healthy children and adolescents that found that PI regulated SS and PT, but there was a weaker correlation between TK and PI [25]. The lumbar spine seems to function as the absorber between the thoracic spine and pelvis to lessen the influence of the alignment on its counterpart.

### Three types of thoracic AIS by cluster analysis

In our cohort, the significant factors for a decrease in TK were an increase in SS and decrease in max-LL, and thoracic AIS could be classified into three types based on SS and max-LL. (Fig. 3).

Type 1 is thoracic AIS that manifests as flat global sagittal alignment with low SS and low max-LL. This type is hypokyphotic thoracic scoliosis with an average TK of 15°. The LL is small, seemingly adapting to this small TK. Type 2 is thoracic AIS that manifests with high SS and low max-LL and has very small TK, with an average of 6°. This type corresponds to the sagittal thoracic modifier “minus” in the Lenke classification. Type 3 is thoracic AIS that manifests with high SS and high max-LL and exhibits undulating sagittal alignment at an average TK of 23°. This type has a large LL that looks well balanced with a large SS. Its larger TK seems to adapt to the large LL. Abelin-Genevois classified AIS sagittal alignment into four categories and reported that a fraction of patients had the curve pattern of thoracolumbar lordosis with a proximally shifted inflection point [11]. The reason why we did not find this type among our patients is unclear, but the reason may be attributable to racial differences, minor but nonetheless present difference in the pelvic shape between the two studies, or possibly a prevalence too low to form another cluster category in our study.

Type 2 has a unique sagittal profile. The lumbar lordosis seems disproportionately small compared to the large sacral slope. The IP SVA was significantly anterior, while the levels of the IP and C7 SVA remained similar compared to those in the other types (Fig. 4). In the comparison between Type 2 and Type 3, both had similar PI, SS, PT, level of IP, coronal Cobb angle, and C7 SVA, but significant differences were found in LL, max LL, TK, and IP SVA (Fig. 4). Interestingly, despite similar sacropelvic alignment and C7 SVA values, Type 2 patients presented with largely different sagittal profiles from Type 3 patients. We tried to find the theoretical reason for this difference, which may lie in the flexibility between the thoracic spine and thoracolumbar/lumbar spine. The more flexible segment should function as the compensator for ergonomic alignment. Here, two possible hypotheses for this mechanism are presented. One is due to the stiffer thoracic spine, and the other is due to the stiffer lumbar spine.

One hypothesis is that stiff lordoscoliosis or hypokyphotic scoliosis in the thoracic spine influences the shape of LL or IP SVA. SS is said to develop and stabilize as the individual starts to walk in his or her early childhood, while PI and LL continue developing until the teenage years [28]. As the child grows, sufficient LL for the SS should develop in the patients of Type 2. However, stiff lordoscoliosis manifests in adolescence, and this change would shift the C7 SVA posteriorly. Between the stabilized SS and the stiff TK, the more flexible thoracolumbar/lumbar spine reacts as a buffer to shift the IP-SVA anteriorly by becoming less lordotic.

The other hypothesis is that a sufficiently large LL cannot develop, so TK compensates for the anterior shift of the IP SVA by becoming as flat as possible to achieve the standard C7 SVA. If LL is defined by PI far more strongly than by SS, the aforementioned gap in the timing of maturity between LL/PI and SS may be the key to explaining why a sufficient large LL cannot be achieved. That is, if the immature small PI defines the small LL, this LL would not be large enough for the SS, which has already fully developed in early childhood. This insufficient LL would result in an anterior shift of the IP SVA, and this shift would require compensation by the uniquely flat TK to hold the C7 SVA within the standard range.

In both of these hypotheses, the chronological gap of the maturity of PI/LL and SS plays a role and should be considered. This gap was mentioned by MacThiong et al. [2, 28]. According to them, SS stabilizes just after the initiation of bipedal walking in early childhood and does not significantly change toward adulthood. In contrast, he also states that PT gradually enlarges to manage the growing and posteriorly shifting mechanical load from the center of gravity over the growth period. Following this development of PT, PI also grows larger under the formula PI = PT + SS, driving the increase in LL as well. As such, this gap in maturity among several sagittal parameters seems to occur over a decade. This would be long enough to induce a unique compensatory cascade for balanced sagittal alignment in developing children, as proposed in our hypotheses.

The limitations of this study are as follows. The included patients ranged in age from preadolescence to young adulthood. This age range varies in many aspects, including growth phase and spinal stiffness. For example, spinal stiffness may influence radiographic appearance, especially when the compensatory mechanism is thought to influence the deformity. The number of total cases included was not large enough to stratify the cases based on age, but ideally, we should perform age stratification as more cases become available. The other limitation is that we assessed the curve only on lateral X-ray images. It has been reported that thoracic kyphosis is overestimated on 2D images compared to 3D images because of vertebral rotation [29]. This reflects that our thoracic AIS patients may have less TK or TLK or a higher inflection point than if these parameters were measured on 3D images. This susceptibility to rotation may also suggest that the angular measurements made with lateral X-ray images can easily vary, even in the same subject, if the X-ray beams are not shot exactly in the true lateral direction.

## Conclusions

In thoracic AIS, a decrease in TK corresponded to an increase in SS or a decrease in max-LL, where max-LL was defined as the lordotic Cobb angle measured from the S1 endplate to the inflection endplate between thoracic kyphosis and lumbar lordosis. By analyzing their X-ray images, the thoracic AIS patients were classified into three types based on SS and max-LL. As represented by one of these three types, the existence of patients with large SS, small LL, and very small TK is clarified. Patients with thoracic hypokyphosis are known to be at risk for clinical problems with pulmonary function or lumbar degeneration, and special attention should be given to restoring the thoracic sagittal alignment and correcting the coronal deformities of these patients.

**Table 1:** Radiographic parameters

Parameter	mean	SD
Cobb angle of thoracic scoliosis	40.9˚	16.5˚
Cobb angle of lumbar scoliosis (L1-S1)	16.3˚	8.6˚
Thoracic Kyphosis (TK)	16.1˚	9.1˚
Lumbar Lordosis (LL)	53.0˚	11.0˚
Max-LL^a^	56.4˚	10.8˚
C7 sagittal vertical axis (C7 SVA)	-15.4 mm	24.1 mm
Pelvic Incidence (PI)	48.5˚	11.6˚
Pelvic Tilt (PT)	8.8˚	7.2˚
Sacral Slope (SS)	39.5˚	8.8˚
Inflection Point^b^	-0.5	1.3
Inflection Point SVA (IP SVA)^c^	5.5 mm	17.0 mm

^a^max-LL was defined as the maximum lordotic Cobb angle from S1, which is measured from the upper end plate of S1 to the upper endplate of the vertebra at the inflection point between thoracic kyphosis and lumbar lordosis

^b^Inflection point was defined as the junction between the kyphosis and lordosis, and was denominated 0 when this point was at L1, and positive number was given when this goes down caudally (i.e., +1 at L2, +2 at L3, -1 at T12)

^c^IP SVA is SVA of the inflection point, measured as the horizontal distance from the posterosuperior corner of S1 vertebral body to the inflection point

**Table 2:** Stepwise and multivariate logistic regression analysis to explain the small TK

Stepwise analysis			Multivariate logistic regression analysis			
Parameter	Estimate	*p* value	Liklihood rate	*p* value	Odds ratio	95%CI
Inflection Point	0	0.89738	―			
IP SVA	0.01984	0.26577	1.2736008	0.2591	1.02	0.99-1.06
C7 SVA	0	0.28255	―			
PI	0	0.54894	―			
PT	0	0.65637	―			
SS	0.11811	0.02579	6.02394353	0.0141*	1.12	1.01-1.25
Max LL	-0.09688	0.01484	7.2965093	0.0069*	0.91	0.84-0.98
LL	0	0.38897	―			
Cobb angle	0	0.98805	―

**Table 3:** Comparison with historical data of AIS and healthy subjects

	Population	Sampe size	Age	PI	PT	SS	TK	LL
Current	T-AIS	83	15.9	48.5	8.8	39.5	16.1	53.0
Yong^8^	T-AIS	95	14.2	44.2	9.2	35.1	15.7	48.5
Mac-Thiong^9^	AIS(King3)	42	13.4	57.3	8.4	47.9	20.3	40.8
Upasani^10^	AIS (Lenke1)	53	14.5	55.5	11.5	45.8	18.9	60.5
Lonner^11^	AIS(Black)	115	15.0	56.0	13.9	42.5	24.7	63.6
Lonner^11^	AIS(White)	421	14.8	52.5	10.8	42.2	22.9	59.1
Yong	Healthy girls	33	13.6	44.6	11.3	33.3	20.8	49.3
Upasani^10^	Healthy individuals	50	13.5	45.5	8.4	37.1	27.9	55.1

## Data Availability

The datasets used in the current study are available from the corresponding author upon reasonable request.

## Abbreviations

AIS: Adolescent idiopathic scoliosis
TK: Thoracic kyphosis
LL: Lumbar lordosis
SVA: Sagittal vertical axis
PI: Pelvic incidence
PT: Pelvic tilt
SS: Sacral slope
IP: Inflection point
